# Effect of the Ultrasonic Surface Rolling Process on the Fretting Fatigue Behavior of Ti-6Al-4V Alloy

**DOI:** 10.3390/ma10070833

**Published:** 2017-07-20

**Authors:** Chengsong Liu, Daoxin Liu, Xiaohua Zhang, Shouming Yu, Weidong Zhao

**Affiliations:** Corrosion and Protection Research Laboratory, Northwestern Polytechnical University, 127 You Yi Xi Road, Xi’an 710072, China; liu3307778@126.com (C.L.); yhzhangxh@163.com (X.Z.); shoumingyu@163.com (S.Y.); zhaoweid@mail.nwpu.edu.cn (W.Z.)

**Keywords:** Ti-6Al-4V alloy, ultrasonic surface rolling process (USRP), compressive residual stress, surface work-hardening, fretting fatigue (FF)

## Abstract

The effect of the ultrasonic surface rolling process (USRP) on the rotary bending fretting fatigue (FF) of Ti-6Al-4V alloy was investigated. The reason for the USRP’s ability to improve the FF resistance of Ti-6Al-4V alloy was studied. The results revealed that the USRP induced a compressive residual stress field with a depth of 530 μm and a maximum residual stress of −930 MPa. Moreover, the surface micro-hardness of the USRP sample was significantly higher than that of the untreated base material (BM) sample, and the USRP yielded a 72.7% increase in the FF limit of the alloy. These further enhanced fatigue properties contributed mainly to the compressive residual stress field with large numerical value and deep distribution, which could effectively suppress FF crack initiation and early propagation. The USRP-induced surface work-hardening had only a minor impact on the FF resistance.

## 1. Introduction

Titanium alloys have been widely used in the manufacture of aero-engine compressor blades and disks owing to their low density, high specific strength, excellent corrosion resistance, and ability to operate at elevated temperatures. However, these alloys suffer from drawbacks such as low wear resistance, high notch sensitivity, and high susceptibility to fretting fatigue (FF). The FF-related damage becomes the main failure mode of the blade/disk dovetail joint attachments, creating a potential safety hazard when titanium alloy components are operated under similar working conditions [1]. Thus, surface engineering techniques for improving the FF performance of titanium alloys have gained considerable attention [2,3,4]. Among these surface treatment methods, conventional shot peening (SP) is generally acknowledged as one of the most effective methods for improving the FF resistance of titanium alloys [5,6]. Recent studies have reported that laser peening [7], low plasticity burnishing (LPB) [8], and deep rolling (DR) [9] can significantly improve the FF resistance of metallic materials. These methods typically yield superior FF resistance to that obtained via conventional SP.

In recent years, the USRP technique, which combines the advantages of DR, ultrasonic impact treatment [10], and LPB, has shown a number of advantages [11,12,13,14]. The principle of the USRP is that electrical energy can be transformed into mechanical energy via piezoelectric ceramics. The mechanical energy is amplified and aggregated by the ultrasonic horn, resulting in a high-power ultrasonic wave, which drives the impact tool ball that strikes and rolls along the metal surface at high velocities. These repeated and high-frequency strikes lead to severe plastic deformation, which induces a deeper, higher-magnitude residual compressive stress field than that induced by conventional SP [11,14]. Compared with conventional SP, the USRP is more effective in reducing the surface damage and yields a relatively lower surface roughness, owing to the free-rolling state of, and liquid lubrication applied to, the impact tool ball [11,12]. Similarly, the USRP enables the generation of surface hardness layers via severe plastic deformation caused by grain refinement and dislocation multiplication [11,12,15]. Nowadays, this technique has been used increasingly to improve the plain fatigue and wear resistance performance of mechanical components [11,16,17,18,19]. However, to date, little research has been done on the effect of the USRP on the anti-FF behavior of Ti-6Al-4V alloy. FF failure always results from concurrent fatigue damage and fretting wear [3,4]. The effect of conventional SP on the FF behavior of metallic materials has been clarified [5,6,7]. The conventional SP technique has three main factors to improve the FF performance of materials: compressive residual stress (labeled σ_r_), surface work-hardening (labeled H), and increase of surface roughness. The improvement of anti-FF performance by conventional SP is attributed mainly to the compressive residual stress generated on the surface, which effectively suppresses the initiation and early propagation of fatigue cracks. Conventional SP also leads to work-hardening beneath the surface, thereby preventing crack initiation. The increase of surface roughness induced by conventional SP can decrease the fretting contact area and furthermore reduce the probability of FF crack initiation [6]. However, the effect of USRP on the improving the FF resistance of Ti-6Al-4V alloy remains unclear, and the mechanism is not yet established.

In the present work, the USRP technique was employed to strengthen the surface of Ti-6Al-4V alloy. As a result, a surface microstructure refinement layer with high surface hardness, low surface roughness, and high compressive residual stress was obtained. The effect of USRP on the FF behavior and the main factors for the USRP improving the FF resistance of Ti-6Al-4V alloy were also investigated.

## 2. Experimental Procedures

**Figure 1:**
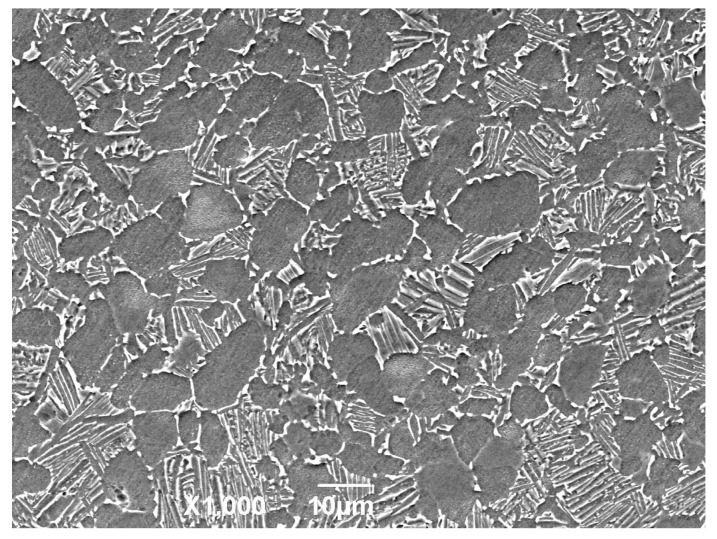
The microstructure of the annealed-state Ti-6Al-4V alloy.

The test material considered in this investigation was annealed-state Ti-6Al-4V alloy with the following chemical composition (mass %): Al-6.7, V-4.2, Fe-0.1, C-0.03, N-0.015, H-0.03, O-0.14, and balance Ti. The material was vacuum-annealed at 890 °C for 1 h and then cooled in air. The microstructure consisted of inter-connected equiaxed α-grains and α + β colonies (transformed β), as shown in Figure 1. This alloy has a yield strength of 1010 MPa, a tensile strength of 1080 MPa, an elongation of 14%, and a section shrinkage of 41%. FF samples and fretting pads were obtained from this annealed Ti-6Al-4V alloy.

**Figure 2:**
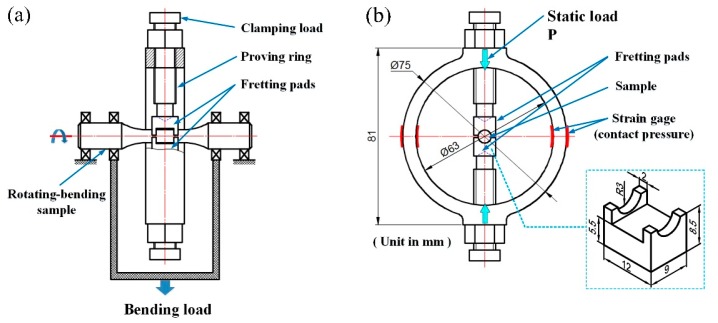
Schematic illustrations of the FF test apparatus (**a**) Principle of the rotary bending FF test; (**b**) shape and dimensions of the proving ring.

This study used a modified PQ-6 type rotary bending fatigue tester as the experimental apparatus. A self-made fretting generation system, attached to the rotating bending fatigue test machine, was designed and developed to investigate the FF behavior of the Ti-6Al-4V samples while in contact with two Ti-6Al-4V fretting pads. The schematic of the FF test apparatus is shown in Figure 2. The system (including test sample, fretting pads, and probing ring) was rotated simultaneously, as shown in Figure 2a. The contact point between the fretting pad and the FF sample was arc surface to arc surface, with a contact area of about 33.70 mm^2^. The surfaces of the FF sample and the fretting pad were mechanically polished with a smooth surface (Ra < 0.3 μm). In addition, the contact pressure was applied to the fretting pad by adjusting screws attached to the probing ring and was maintained at 150 MPa using a tension sensor, as shown in Figure 2b. Laboratory-scale FF tests (see Reference [20] for size of the fatigue samples) were conducted at room temperature, applying the sinusoidal circle stress at a constant stress ratio (σ_min_/σ_max_) of −1 and a uniform frequency of 35 Hz.

**Figure 3:**
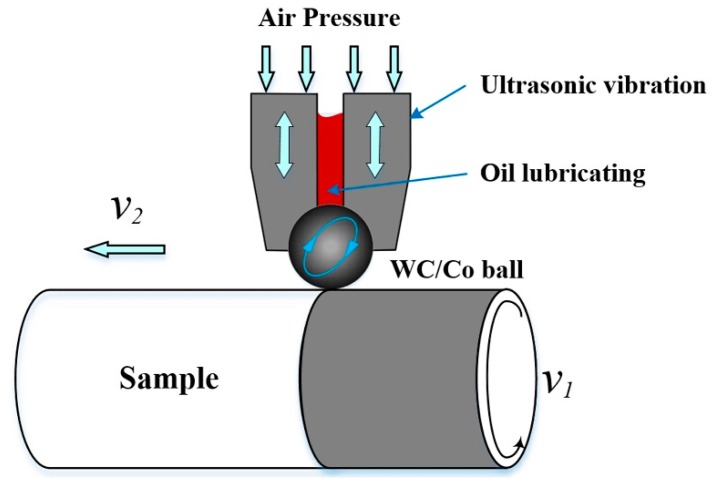
Schematic illustration of USRP set-up.

The USRP experiments were performed on a self-built platform based on a conventional lathe. The schematic diagram of the USRP set-up is shown in Figure 3. A scrollable rolling WC/Co ball was firmly attached to the ultrasonic apparatus, and then allowed to roll and strike the surface of samples at thousands of strikes per second under a static force. After the USRP, a microstructure-refined surface layer was generated at high strains and high strain rates. The WC/Co ball had a hardness, surface roughness, and diameter of 80 HRC, Ra 0.1 μm, and 14 mm, respectively. The basic USRP parameters are presented in Table 1.

The surface roughness of all samples was determined by using a TR-300 surface roughness tester (Beijing Timesun Measurement and Control Technology Co. Ltd., Beijing, China). The microstructures, the corresponding surface morphologies, and the fracture features were examined on a JSM-6390 scanning electron microscope (SEM, JOEL Ltd., Tokyo, Japan). A ZEISS MERLIN Compact SEM (Carl Zeiss AG, Oberkochen, Germany) was used to analyze the cross-sectional microstructure and to obtain electron backscatter diffraction (EBSD). Samples for EBSD examination were prepared using a Gatan Ilion^+^ Broad Ion Beam (BIB, Gatan, Inc., Pleasanton, CA, USA) system. The micro-hardness of near surface regions was measured (load: 25 g, dwell time: 20 s) using a MHV-1000 micro-hardness tester (Sinowon Innovation Metrology Manufacture Limited, Dongguan, China) equipped with a Knoop diamond indenter. Each test was repeated five times and the hardness was taken as the average of the five values. Residual stress values along the axial direction were measured using an X-ray diffractometer (PROTO LXRD MG 2001, PROTO Manufacturing Ltd., Oldcastle, ON, Canada), employing the classical sin^2^Ψ method with Cu-Kα radiation at the {213} plane of the hexagonal α-phase. In the case of α-Ti, the X-ray diffraction (XRD) scans were performed over 2θ ranging from 134° to 148°. The principle of XRD stress determination can be found in Reference [21]. To determine the in-depth residual stress distributions, the materials were successively removed by a mixture of HNO_3_ and HF solution and subsequently measured via XRD.

In order to study the effect mechanism of USRP treatment on FF behavior of Ti-6Al-4V alloy, the separation of influencing factors (such as compressive residual stress, surface work-hardening, and increase of surface roughness) was carried out. However, the average surface roughness value of USRP samples is 0.108 μm, which is smaller than that of the base materials (BM) samples (0.218 μm), as shown in Table 2. In contrast with the mechanically polished BM sample surface, the USRP sample shows a smoother and a more compact surface morphology. Therefore, the effect of surface roughening is negligible in this paper. Based on the author’s research [6,22], the influencing factors of surface work-hardening and compressive residual stress were separated using the following method. The USRP samples were annealed at 500 °C for 1 h (A) and then slightly etched for about 6 s with a mixture of HF and HNO_3_ (HF:HNO_3_:H_2_O = 2:1:1) to remove the effect of the surface oxide film (the resulting sample is referred to as USRP + A). The removal rate of the thin layer was about 20 μm/min. The USRP-induced compressive residual stress of Ti-6Al-4V alloy may be relaxed significantly at the annealing temperature. In addition, the effects of work hardening can be retained to some extent. The average surface roughness value of USRP+A samples is 0.187 μm.

## 3. Results and Discussion

### 3.1. Microstructure of the USRP Surface Layer

**Figure 4:**
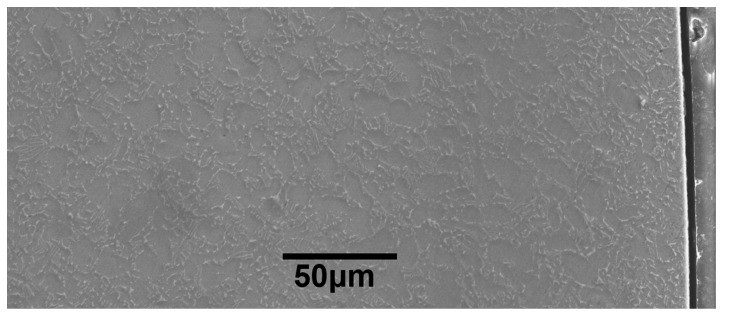
The cross-sectional microstructure of the USRP sample.

**Figure 5:**
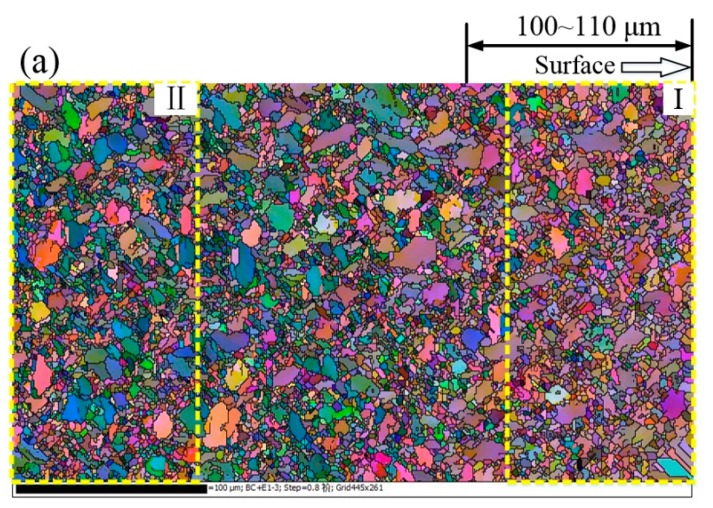

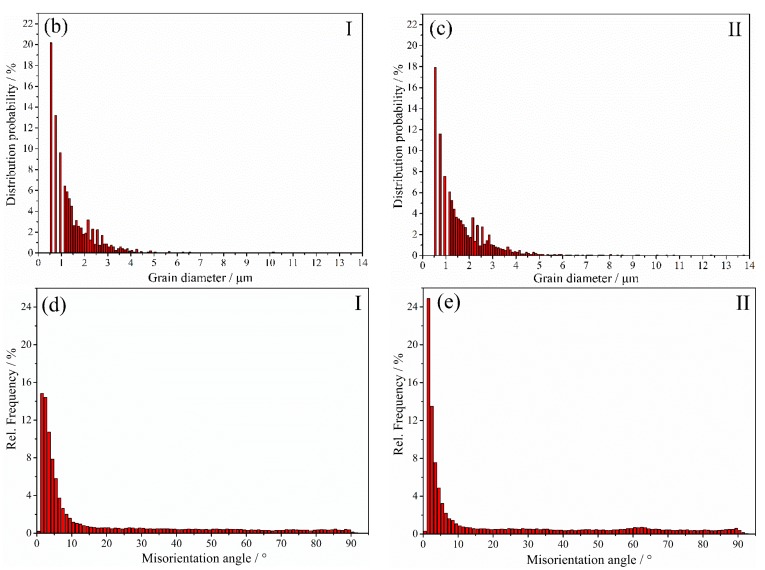
Cross-sectional EBSD map of Ti-6Al-4V alloy treated by the USRP (**a**); and corresponding grain diameter distribution (**b**,**c**) and misorientation angle distribution (**d**,**e**): Ι zone—the near surface; ΙΙ zone—the sub-surface.

The cross-sectional microstructure of the USRP sample is shown in Figure 4. It can be observed that the surface microstructure refinement of the USRP sample is not obvious in the treatment parameters from SEM analysis. Figure 5 shows the microstructure, grain diameter distribution and misorientation angle distribution in the strengthened layer induced by the USRP with the help of EBSD characterization. It can be seen from Figure 5a that the surface microstructure refinement layer occurs at depths of up to 100~110 μm. The average grain diameter of the near surface layer (Ι zone) is about 1.46 μm (Figure 5b), which is smaller than that of the sub-surface layer (ΙΙ zone) which measures about 1.68 μm (Figure 5c). Moreover, the statistical data show that the distribution probability of grain diameters within the range of 0~3 μm in the near surface layer is 92.3%, which is slightly higher than the sub-surface layer at 89.1%. This means that the microstructure of the surface layer is slightly refined after the USRP. The refined microstructure facilitates the reinforcement of hardness, wear resistance and compressive residual stress. The length and density of the low angle boundary (with misorientation angle <15°) calculated by EBSD analysis can quantitatively reflect the microscopic deformation [23]. The misorientation angle distribution data suggest a slight increase in the percentage of low angle boundary from 64.1% in the sub-surface layer (Figure 5e) to 68.5% in the near surface layer (Figure 5d). This indicates that microcoscopic deformation occurs in the surface layer. In addition, the increased number of the low angle grain boundaries can prevent dislocation from slipping, so as to improve the fatigue strength of the material [24].

### 3.2. Micro-Hardness Distributions along the Cross-Section

**Figure 6:**
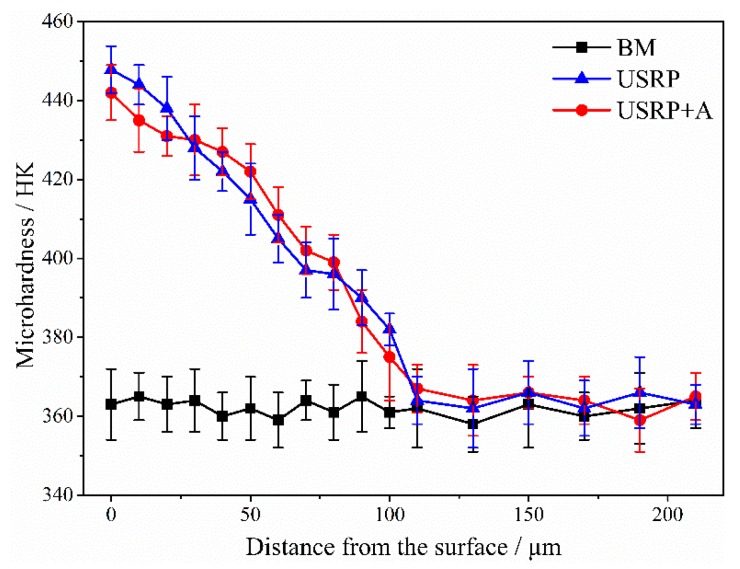
The results of micro-hardness distribution along the cross-section of samples with different treatments.

The micro-hardness distributions of BM, USRP + A, and USRP sample cross-sections are shown in Figure 6. As the figure shows, a uniform Knoop micro-hardness of ~360 HK is obtained for the unprocessed BM sample. In contrast, a micro-hardness distribution, characterized by a gradient distribution where the hardness decreases with increasing distance from the top surface of the material, is obtained for the USRP sample. The hardness (450 HK) at the top surface of the USRP sample is 25% higher than that of the BM sample. In addition, the ~110 μm deep work-hardening layer induced in the USRP sample is basically consistent with the depth of the refined structure layer shown in Figure 5. The work-hardening degree of the USRP + A sample is close to that of the USRP sample, indicating that the configuration of surface dislocations remains almost unchanged [6,22].

### 3.3. Distribution of Residual Stresses

**Figure 7:**
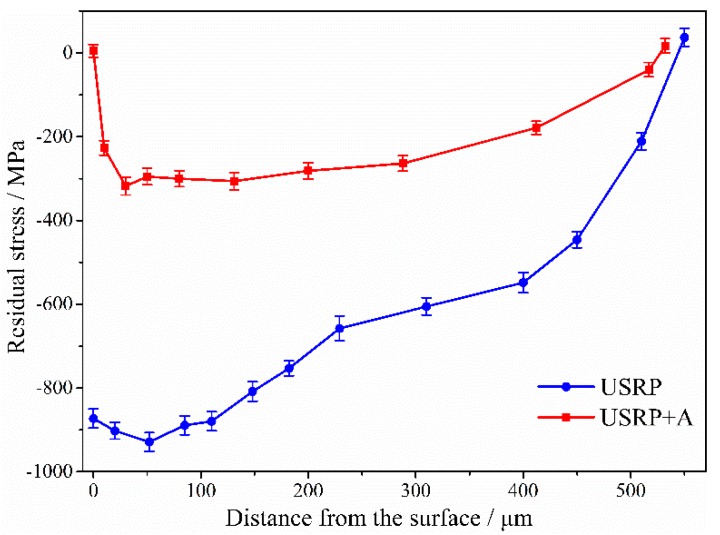
The results of axial residual stress distribution along the cross-section of samples with different treatments.

The depth distributions of the axial compressive residual stresses associated with different samples are shown in Figure 7. A compressive residual stress field with a depth of ~530 μm was induced in the surface layer by USRP, and a compressive residual stress of ~870 MPa occurs at the surface. In addition, after USRP, a maximum compressive residual stress of ~930 MPa occurs at a depth of ~50 μm. The value and depth of the USRP-induced compressive residual stress field are higher than those induced by conventional SP [7]. The surface and sub-surface compressive residual stresses are almost completely relaxed and significantly relaxed, respectively, after annealing at 500 °C for 1 h. However, a 200~300 MPa compressive residual stress is still retained in the 400 μm deep layer for the USRP + A sample.

### 3.4. FF Test Results

**Figure 8:**
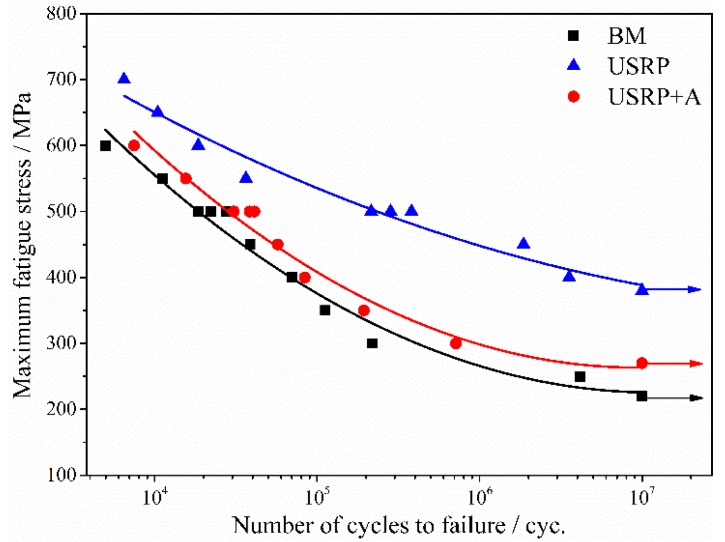
Stress/life (S-N) curves for BM, USRP + A and USRP samples.

The stress/life (S/N) fatigue behavior of Ti-6Al-4V alloy is shown in Figure 8 in terms of the maximum fatigue stress (σ_max_) as a function of the number of cycles to failure (N_f_) for the USRP + A and USRP samples, as compared to the untreated BM sample. The figure shows that the fatigue strength of the alloy increases significantly, with the USRP, from the low cycle fatigue (LCF) to the high cycle fatigue (HCF) regimes. The fatigue limit of the the USRP sample (about 380 MPa) is ~72.7% higher than that of the BM sample (about 220 MPa). Moreover, the improvement effect of USRP on the FF strength is more remarkable than that reported in studies investigating the effect of mechanical surface treatment methods on plain fatigue [16,17,18]. The FF limit of the USRP sample annealed at 500 °C for 1 h (about 270 MPa) is 28.9% lower than that of the USRP sample, but still 22.7% higher than that of the BM sample. However, the fatigue limit values of the BM, USRP + A, and the USRP samples shown in Figure 8 correspond to only one unbroken sample, rather than values obtained through a staircase method. Therefore, these non-statistically evaluated S/N curves must be interpreted with caution.

**Figure 9:**
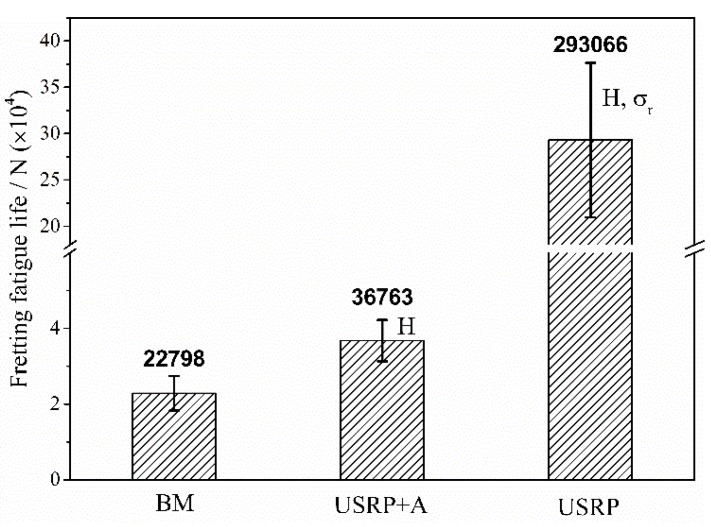
Fretting fatigue lives of Ti-6Al-4V alloy with different surface treatments at a maximum stress level of 500 MPa (H-surface work-hardening, σ_r_-compressive residual stress).

The average FF life of BM, USRP + A, and USRP samples (σ_max_ = 500 MPa), which were obtained from three parallel tests for each treatment, is presented in Figure 9. The FF life of USRP sample is 12.9-fold greater than that of the BM sample, which means the FF resistance of Ti-6Al-4V alloy improves significantly with the USRP. The USRP + A sample shows a significant decrease in the FF resistance of the USRP sample. However, the FF life of the USRP + A sample is still 61.3% higher than that of the BM sample.

Figure 9 also shows the main USRP effects influencing the FF life associated with each surface treatment. The work-hardening degree of the USRP + A sample is almost the same as that of the USRP sample (Figure 6). Annealing the USRP sample at 500 °C for 1 h results in considerable relaxation of the compressive residual stress (Figure 7) and the corresponding FF life (36,763) is ~87.5% lower than the pre-annealing value (293,066). Therefore, the USRP-induced surface compressive residual stress plays a key role in improving the anti-FF performance of Ti-6Al-4V alloy. The effect of the USRP-induced compressive residual stress on improving the anti-FF performance of Ti-6Al-4V alloy is attributed mainly to the inhibition of FF crack initiation and early propagation [6,25]. A previous study demonstrated that compressive residual stress field is also beneficial to retard the fretting wear and to promote the crack closure [26]. However, the FF life of the USRP + A sample is still 61.3% higher than that of the BM sample. This can be attributed to the synergy between surface work-hardening (Figure 6) and the compressive residual stress remaining after the annealing treatment (Figure 7). Compared with the effect of the compressive residual stress field, surface work-hardening plays a secondary role in improving the FF resistance of the alloy. Surface work-hardening and microstructure refinement can improve the wear resistance at the fretting contact region of the titanium alloy [22] and prevent fatigue crack initiation [17], thereby improving the FF crack-initiation life. Moreover, since the yield strength ratio (σ_s_/σ_b_) of the titanium alloy is relatively higher than that of other materials, the effect of surface work-hardening is not significant. Hence, surface work-hardening plays a secondary role (Figure 9) in improving the corresponding FF resistance.

Furthermore, as shown in Figure 8, the improvement effect of the USRP on the FF resistance of Ti-6Al-4V alloy is more significant under the low-cycle stress HCF regime than that of the high-cycle stress LCF regime. This agrees with findings showing that conventional SP effects improve the FF resistance of Ti-6Al-4V alloy [7,26]. This behavior may be related to the role of crack initiation and propagation. FF life is mainly controlled by the crack initiation process in the HCF regime. The USRP-induced compressive residual stress can effectively prevent crack initiation and premature propagation. Therefore, the improvement of FF life for Ti-6Al-4V alloy after the USRP is remarkable in the HCF regime. Meanwhile, more relaxation of compressive residual stress, which can be attributed to the fatigue loading, was observed in the high-cycle stress LCF regime [27,28]. Hence, the improvement in FF life observed for USRP samples is not remarkable in the LCF regime.

**Figure 10:**
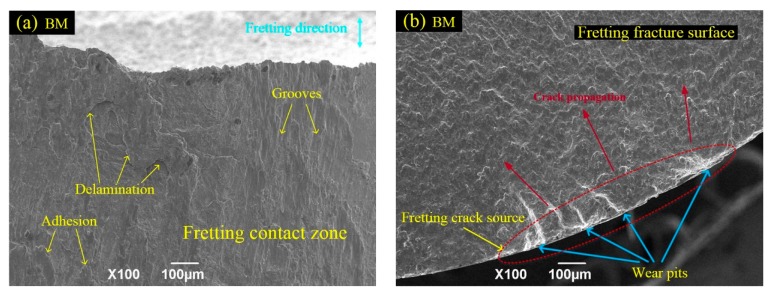

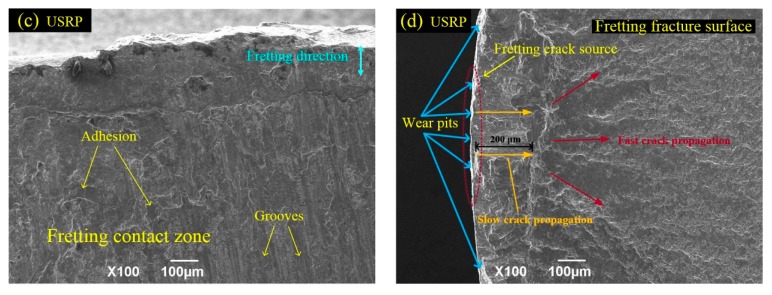
Morphologies of the fretting contact zones and the fracture surfaces of BM and USRP samples: (**a**,**b**) BM sample; (**c**,**d**) USRP sample (maximum stress level σ_max_ = 500 MPa).

Figure 10 shows the morphologies of the fretting contact zones and FF fracture surfaces of the BM and USRP samples at a maximum stress level of 500 MPa. Severe surface wear damage occurs in the fretting contact zones of both the BM sample (Figure 10a) and the USRP sample (Figure 10c). The BM sample exhibits composite failure characterized by the occurrence of abrasive wear, fatigue wear (local delamination), and adhesion wear. Whereas the USRP sample (where fatigue delamination was suppressed) exhibits abrasive wear and adhesive wear characteristics. The crack initiates from wear pits in the fretting region of the BM sample (Figure 10b). These wear pits result from the (i) vibration-induced shedding of fretting debris from the surface of the FF sample; or (ii) from the transfer of some adhesive material to fretting pads, owing to intense co-friction of the FF sample and fretting pads. The presence of these pits leads to severe stress concentration on the FF sample, which promotes crack initiation and propagation [29,30]. The FF sample of BM exhibits a multi-crack source fatigue fracture and the fatigue life is dominated by the initiation stage. After crack initiation, the cracks expand immediately to the interior, thereby resulting in early fatigue fracture (Figure 10b). The fatigue crack of the USRP sample also initiates from wear pits in the fretting region, but propagates slowly for a distance of ~200 μm and then rapidly into the interior (Figure 10d). This may have resulted from the larger magnitude and depth of the compressive residual stress on the USRP sample surface layer, compared with that on the untreated BM sample (Figure 7). The presence of compressive residual stress can partially offset the applied alternating load and the shear stress in the contact zone. This retards the initiation and early propagation of the FF crack and prolongs the crack propagation life associated with the first stage [25]. In addition, the USRP-induced surface work-hardening results in the improvement of surface wear resistance. The benefits offered by the USRP during FF tests can be attributed to the synergistic effects of the compressive residual stress (the main influence) and the surface work-hardening (the secondary influence).

## 4. Conclusions

The results show that the USRP induced a compressive residual stress field, with a depth of 530 μm, and a maximum compressive residual stress of 930 MPa. Furthermore, the surface micro-hardness of the USRP sample is significantly higher (25%) than that of the untreated BM sample. The FF limit of Ti-6Al-4V alloy is increased by 72.7% after the USRP. The USRP-induced compressive residual stress plays a predominant role in improving the FF resistance of Ti-6Al-4V alloy by retarding FF crack initiation and premature propagation, whereas the surface work-hardening has a secondary influence. The fatigue cracks of the BM and USRP samples both initiate from wear pits in the fretting region.

## Figures and Tables

**Table 1:** The basic USRP parameters.

Ultrasonic Vibration Frequency (kHz)	20
Static force (N)	600
Ultrasonic vibration amplitude (μm)	10
Lathe rotational speed (rev/min)	120
Feeding rate (mm/rev)	0.1

**Table 2:** The surface morphologies and surface roughness (Ra) of BM and USRP samples.

Samples	Surface (× 500)	Surface Roughness Ra
BM	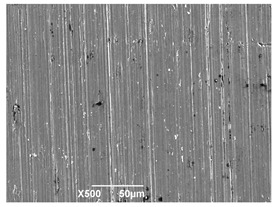	0.218 μm
USRP	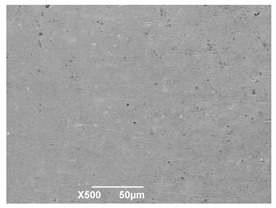	0.108 μm
